# Synergistic Therapy for Graves’ Ophthalmopathy-Associated Eyelid Retraction: Steroid, 5-FU, and Botulinum Neurotoxin a Combination

**DOI:** 10.3390/jcm13103012

**Published:** 2024-05-20

**Authors:** Yuri Kim, Helen Lew

**Affiliations:** Department of Ophthalmology, Bundang CHA Medical Center, CHA University, Seongnam 13596, Republic of Korea; a206014@chamc.co.kr

**Keywords:** botulinum neurotoxin, Graves’ ophthalmopathy, upper eyelid retraction

## Abstract

**Background:** Graves’ ophthalmopathy (GO) is characterized by upper eyelid retraction (UER), the most prevalent clinical sign. We aimed to assess the clinical efficacy of a multimodal combination of steroids, 5-fluorouracil (5-FU), and botulinum neurotoxin A (BoNT-A) injections in managing UER with GO and analyze the clinical factors in relation to the injection response. **Methods:** A total of 37 eyes from 23 patients were enrolled for UER with GO. At the endocrinology clinic, the patients were referred to the ophthalmology clinic after taking antithyroid medication for an average of 5.76 months (13 patients), while 10 patients were initially diagnosed with GO and referred to the endocrinology clinic for management of the thyroid hormone function. They performed an orbital computed tomography (CT) scan and measured the cross-sectional area of the orbit, orbital fat, and each extra ocular muscle (EOM) except for the inferior oblique muscle 4 mm behind the eyeball. Each of the EOMs and orbital fat were calculated as a ratio to the total orbit area. A total of 0.1 cc of triamcinolone (40 mg/mL), dexamethasone (5 mg/mL), 5-FU, and BoNT-A (2.5 units) was injected transconjunctivally. Medical records were examined and photographs were utilized to assess MRD1, inferior palpebral fissure (IPF), and lid lag during down gaze before and after the injection. The patients were divided into two groups: responders (more than 1 mm decrease in MRD1 after injection) and non-responders. During the follow-up period (11.0 ± 11.6 months), any potential adverse effects were monitored. **Results:** CAS decreased from 3.0 ± 0.8 to 1.4 ± 0.5 after the injection, and MRD1 decreased from 5.0 ± 0.9 mm to 4.5 ± 1.3 mm. Sixty percent of the patients were responders. Before and after the injection, the difference between IPF and MRD1 in responders was 0.60 ± 1.10 mm and 0.90 ± 0.90 mm, respectively, whereas, in non-responders, it was −0.57 ± 0.88 mm and −0.15 ± 0.75 mm, respectively. In the responders, pre-injection IPF and FT4 were significantly higher (*p* < 0.05). Responders had a larger EOM cross-sectional area (153.5 ± 18.0 mm^2^), including a larger lateral rectus muscle cross-sectional area (37.6 ± 9.7 mm^2^) than non-responders (132.0 ± 27.9 mm^2^; 29.1 ± 8.1 mm^2^). In responders, the treatment effect on IPF and MRD1 remained consistent at 1.2 ± 3.4 mm and 1.2 ± 1.6 mm, respectively, during the latest follow-up assessment. **Conclusions:** The combination injection of corticosteroids, 5-FU, and BoNT-A would be effective, especially, in patients with hyperthyroidism and an elongated IPF. Additionally, an increase in EOM cross-sectional area on CT, up to 150 mm^2^, may serve as an additional positive indicator for the use of multimodal injections in UER with GO.

## 1. Introduction

Advanced Graves’ ophthalmopathy (GO) could cause ocular pain, edema, and redness of the eyelids, conjunctival chemosis, and congestion; severe GO can lead to exposed keratitis and corneal ulcers [1]. GO is characterized by upper eyelid retraction (UER), the most prevalent clinical sign [2]. The causes of UER are complex, but there may be an increase in the sympathetic tone of Mueller muscles, an increase in levator muscle contraction, and hyperactivity of the levator muscle or Mueller muscle [3,4].

GO varies in disease status by period. In the early or acute phase, acute inflammation by lymphocytes and fibroblasts is the primary cause of ophthalmic tissue edema, whereas, in the chronic phase, fibrosis is known to develop in the tissues surrounding the eye, including the external ocular muscle, ocular fat, lacrimal gland, eyelid, and eyelid muscles [5]. Recently, new treatments for GO have been devised, and the recommended treatment varies based on the disease condition of the patient. For instance, in the early stages of GO, prior to the onset of EOM fibrosis, drug therapy such as steroids, immunosuppressants, and radiation therapy can be beneficial, with steroids inhibiting inflammatory changes reliably [5]. In the late stages of GO, surgical correction is contemplated.

In the case of GO-induced UERs, many patients experience discomfort during this waiting period because they do not undertake surgical treatment until the inflammatory phase of the disease has subsided if it does not pose a threat to their vision. Throughout this time period, numerous UER treatment attempts have been undertaken. Local injections of botulinum toxin A (BoNT-A) and triamcinolone have been reported as treatments for UER. According to Morgenstern et al., BoNT-A is used for eyelid extension by inhibiting acetylcholine secretion in the neuromuscular junction to relax the rhabdomyolysis and smooth muscle, and it can also be used to treat UER in GO patients during the inflammatory phase [2]. BoNT-A injections into the conjunctiva, according to Costa et al., decrease UER and levator muscle function and aesthetically enhance the eyelid shape [6]. Jung et al. and Kim et al. reported that eye peripheral triamcinolone injections reduced edema and congestion in acute GO and that Xu et al.’s repeated triamcinolone injections were also effective in treating UER [7,8,9]. There is also evidence that 5- fluorouracil (5-FU) may be more efficacious in triamcinolone +5-FU injections than triamcinolone injections alone in reducing fibroblastic proliferation [10,11].

In previous studies, injections targeting the conjunctiva, eyelids, and ocular area have shown positive effects in treating UER caused by GO. However, there has been a lack of research on the effects of a multimodal approach. In this study, the authors aimed to analyze the effects of a conjunctiva-administered multimodal injection consisting of four medications: triamcinolone, dexamethasone, 5-FU, and a low dose of BoNT-A.

## 2. Materials and Methods

### 2.1. Patients

This study was retrospectively conducted between January 2015 and July 2021. This study was approved by the Ethics Committee of Bundang CHA Medical Center and all patients signed an informed consent form. The patients fulfilled the following inclusion criteria: mild to moderate GO with features of either unilateral or bilateral lid retraction, stable thyroid function, age of 18 years or older, absence of other local and systemic disease related to eyelid malposition such as blepharospasm, myasthenia gravis, and muscular dystrophy. Exclusion criteria were lack of orbital CT images before injection and a follow-up period less than two months after injection. A total of 37 eyes from 23 patients were enrolled for upper eyelid retraction (UER) with GO. At the endocrinology clinic, the patients had undergone a minimum of three months of treatment with antithyroid medication.

The patients presented with varying thyroid conditions at the time of injection, and, if required, additional treatments such as radiotherapy, oral prednisolone (Solondo, Yuhan, Republic of Korea), and intravenous methylprednisolone (methysol inj., Alvogen, Republic of Korea) were administered prior to the injection. To evaluate the activity and severity of GO, a clinical activity score (CAS) and NOSPECS classification were used. Proptosis was measured by Hertel exophthalmometry (Oculus; Oculus Optik Geraete, Wetzlar, Germany). The amount of eye deviation was checked using the HESS test; presence of central diplopia was checked using the BSV test.

The Image J program (1.5v) was used to analyze the photographs based on MRD1, inferior palpebral fissure (IPF), and lid lag during down gaze before and after the injection. Multimodal injection consisted of four medications: BoNT-A (Meditoxin^®^, Amore Pacific, Seoul, Republic of Korea; 2.5 units), 0.1 cc of triamcinolone acetonide (Triamcinolone^®^, Dong Kwang, Seoul, Republic of Korea; 40 mg/mL), 0.1 cc of dexamethasone disodium phosphate (dexamethasone^®^, Yuhan, Republic of Korea; 5 mg/mL), and 0.1 cc of 5-FU (5-FU^®^, JW Pharmaceutical, Seoul, Republic of Korea; 250 mg/5 mL). It was injected transconjunctivally under topical anesthesia using paracaine (Alcaine, Alcon, Republic of Korea).

The patients were divided into two groups: responders (more than 1 mm decrease in MRD1 after injection) and non-responders (less than 1 mm decrease or no change in MRD1 after injection). During the follow-up period (11.0 ± 11.6 months), any adverse effects were tracked, such as diplopia, high IOP, hemorrhage. The patients underwent an orbital computed tomography (CT) scan and had the cross-sectional area of the orbit and each extra ocular muscle (EOM) measured, except for the inferior oblique muscle, orbital fat 4 mm behind the eyeball. Each of EOMs and orbital fat were calculated as a ratio to the total orbit area.

### 2.2. Statistical Analysis

SPSS for Windows, version 27.0, was used for all statistical analyses (IBM Corp., Armonk, NY, USA). Parameters were compared using the paired *t*-test, Mann–Whitney U test, Kruskal–Wallis test, and chi-square test. A *p* value of less than 0.05 was considered to be statistically significant. Using the chi-square test, we reported dichotomous efficacy as odds ratios (ORs) and continuous efficacy as the mean and their respective 95% confidence intervals (CIs). Using correlation analysis, relationships among categorical variables were examined.

## 3. Results

Out of the 23 patients diagnosed with Graves’ ophthalmopathy (GO)-related upper eyelid retraction (UER), a total of 37 patients received conjunctival multimodal injections and were observed for an average duration of 11.0 ± 11.6 months. Among these patients, there were three males and 20 females, with an average age of 44.0 ± 10.2 years. The average duration of Graves’ disease prevalence was 3.26 ± 5.01 months, and the average duration of GO was 2.30 ± 2.07 months. Out of the total 40 injections, three were administered more than once to the same eye, 14 were administered to both eyes, and nine were administered to one side eye. (Table 1).

**Figure 1:**
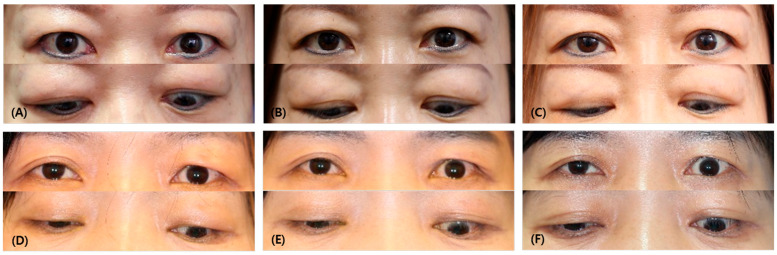
Comparison of photographs of responder and non-responder patients. (**A**) Before injection, (**B**) after 1 month following the injection, (**C**) last follow-up of responder (a 45-year-old female patient who had multimodal injections on both upper eyelids). (**D**) Before injection, (**E**) after 1 month following the injection, (**F**) last follow-up of non-responder (a 57-year-old female patient who had a multimodal injection on her left upper eyelid). Note that in the first month after injection, the IPF decreased by an average of 0.56 ± 1.10 mm in the responder group, while, in the non-responder group, IPF increased by −0.57 ± 0.88 mm (*p* = 0.002). At the last follow-up, the IPF decreased by an average of 1.18 ± 3.42 mm in the responder group, and, in the non-responder group, it increased by −0.39 ± 0.63 mm (*p* = 0.049).

Out of the total number of patients, 14 individuals (60%) showed a positive response to the treatment. In the responder group, there was an average decrease of 0.56 ± 1.10 mm in the IPF measurements during the first month after the injection, while the non-responder group experienced an increase of −0.57 ± 0.88 mm (*p* = 0.002). Comparing the IPF measurements to the last follow-up, the responder group exhibited an average decrease of 1.18 ± 3.42 mm, whereas the non-responder group had an increase of −0.39 ± 0.63 mm (*p* = 0.049). (Figure 1).

At the time of the injection, there were 10 individuals with normal fT4 levels and 12 individuals with elevated fT4 levels. In the responder group, the proportion of fT4 in an elevated state was greater than in the control group (71.4% vs. 25%, *p* = 0.036).

The average CAS score at the time of injection was 3.0 ± 0.8 points, and the NOSPECS was 2.35 ± 1.06; there was no significant difference between the two groups. Three patients with severe eye protrusion and eye movement restrictions before injection had a history of radiation therapy, steroid intravenous therapy in three patients, and oral steroid therapy in three patients. After injection, the average CAS score decreased to 1.4 ± 0.5 points and the NOSPECS decreased to 1.3 ± 0.5; again, there was no significant difference between the two groups (*p* = 0.102, 0.539).

**Figure 2:**
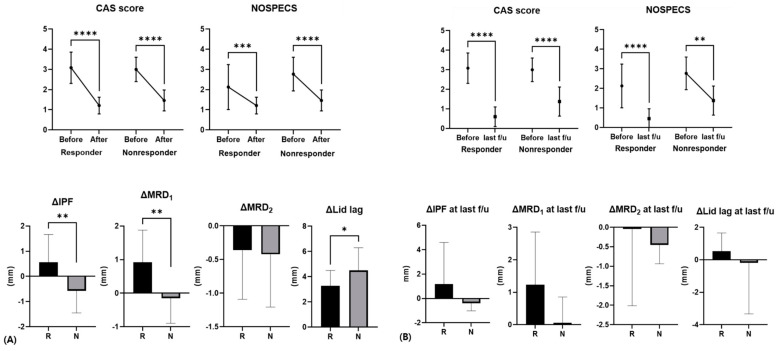
Difference in CAS, NOSPECS score, and eyelid position parameters before and after injection in responders and non-responders. (**A**) After 1 month following injection, (**B**) last follow-up. Average last follow-up period was 11.0 ± 11.6 months. R, responder; N, non-responder (*: *p* < 0.05, **: *p* < 0.01, ***: *p* < 0.001, ****: *p* < 0.0001).

**Figure 3:**
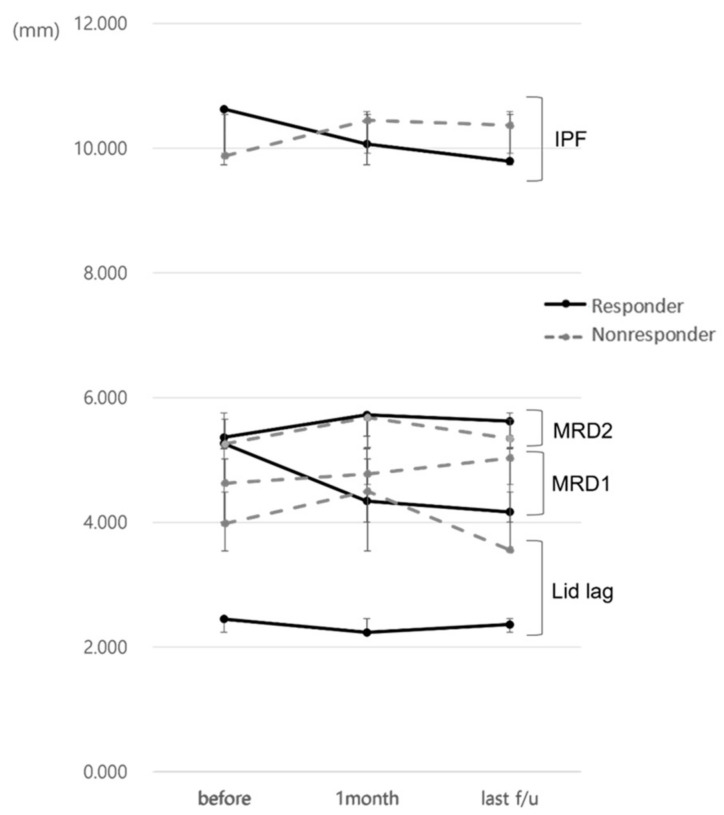
Changes in eyelid position parameters in responders and non-responders during the follow-up.

The responder group had a decrease in MRD1 of more than 1 mm during the first month after injection, while the non-responder group had a decrease of less than 1 mm, no difference, or an increase in MRD1. The IPF before injection was 10.63 ± 1.08 mm and 9.88 ± 0.73 mm in the responder and non-responder group, respectively, with IPF being greater in the responder group (*p* = 0.039) and the IPF difference before and after injection being higher in the responder group (0.56 ± 1.10 mm, −0.57 ± 0.88 mm, *p* = 0.002). The difference between MRD1 levels prior to and following injection was 0.92 ± 0.96 mm, which was also significantly higher in the responder group (−0.15 ± 0.75 mm, *p* < 0.001). There was no statistical significance when compared to the most recent value after injection, but there was a similar tendency (Figure 2 and Figure 3).

**Figure 4:**
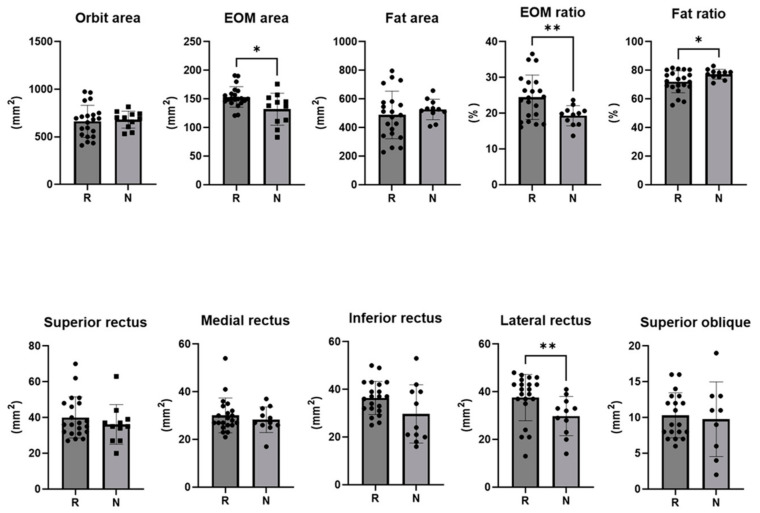
The cross-sectional area of the orbit and each extra ocular muscle, the ratio of the area of the extra ocular muscle to the orbit area, and the ratio of the area of fat to the orbit area in responders and non-responders (*: *p* < 0.05, **: *p* < 0.01).

On the pre-injection CT scan of the responder group, the extraocular muscle area was 153.5 ± 18.0 mm^2^ of the total orbital area, with the lateral rectus muscle area being larger (37.6 ± 9.7 mm^2^). The ratio of extraocular muscle area to total orbital area was 24.48 ± 6.21 mm^2^ in the responder group and 19.30 ± 2.81 mm^2^ in the non-responder group (*p* = 0.003). The ratio of extraocular fat area was 72.01 ± 7.71 mm^2^ in the responder group and 77.31 ± 3.41 mm^2^ in the non-responder group (*p* = 0.012) (Table 2) (Figure 4).

## 4. Discussion

Eyelid retraction related to GO is a challenging condition. Numerous surgical and nonsurgical treatment modalities have been under trial. Management should be based on an individual patient assessment, taking into consideration the disease stage, severity, and clinician experience [12]. Surgery is typically avoided during the active phase of UER caused by GO, unless there is a visual threat. This is because the subjective discomfort experienced by patients sometimes improves as GO progresses. However, in this study, the active phase and chronic phase were not classified separately when administering the multimodal injection. The patients included in the study had a range of thyroid disease phases, from those who were newly diagnosed with Graves’ disease (GD) and visited an ophthalmologist, to those who had been on GD medications for over a year. Some patients received treatment such as oral prednisolone, intravenous methylprednisolone, or radiotherapy before the injection, while others did not receive any treatment. This study specifically recruited patients who were willing to undergo multimodal injection treatment as the primary intervention, given the aesthetic discomfort caused by UER.

In this study, patients’ MRD1 averaged 5.04 ± 0.91 mm, and the responder group, whose MRD1 decreased by more than 1 mm after the injection, comprised 14 of 23 patients (60 percent). Since each group was divided into responder and non-responder based on the change in MRD1 after the injection, the responder group presented higher IPF before the injection. However, the non-responder group showed similar IPF and MRD1 but worse lagophthalmos compared with the responder group after the injection. Given the above, the multimodal injection stretched the levator complex more effectively in the responder group than in the non-responder group, because the non-responder group presented more fibrotic changes in the EOM. One month after the injection, the difference between IPF before and after injection was 0.56 ± 1.10 mm in responders and −0.57 ± 0.88 mm in the non-responders. Considering the long-term effect in the recent photographs, the IPF difference in the responder group was interestingly even greater. Given that the average CAS scores after injection decreased from 3.0 ± 0.8 to 1.4 ± 0.5 points in the total patient population, the reduction of UER and the IPF would be associated with the improvement of CAS. However, there was no significant difference in the change in CAS score between the two groups, and the non-responder group demonstrated less change in IPF due to the fibrotic resistant UER to the multimodal injection.

Botulinum toxin type A (BoNT-A) is widely used as a relaxant for striated muscles such as blepharospasms. BoNT-A and triamcinolone demonstrated similar inhibitory effects on GO orbital fibroblast proliferation and ECM production. BoNT-A showed promise as a treatment for GO by inhibiting TGF-β-induced GO orbital fibroblast activation through the TGF-β/Smad signaling pathway [13]. Morgenstern et al. reported that five units were injected into the skin to weaken the levator muscle, while the conjunctival injection was less efficacious [3]. After injection, MRD1 decreased by 2.35 mm, according to their study. A 39-year-old woman with newly onset proptosis and UER after treatment with anterior levator resection for the progressive ptosis was finally diagnosed with GO and successfully treated with 5 mm of MRD1 with BoNT-A 10 U/0.1 mL [14]. They demonstrated a greater effect of BoNT-A in UER than this study. Morgenstern also argued that injection in the inflammatory stage, the initial active stage of GO, was more effective, and that the surgical effect could be prolonged and the likelihood of surgery progression reduced. In Ozturk Karabulut et al.’s series, the mean normal MRD1 was achieved in 81% of patients [15].

Recently, the effect of incobotulinumtoxin A injection to treat UER in GO has been reported to be as effective as abotulinumtoxin A, whether they applied the toxin transconjunctivaly or transcutaneously [16]. They modified the dose of injection from 2.5 U per eye to 15 U by applying 2.5 U of diluted toxin for each mm of eyelid lowering based on MRD1. After 6 weeks, they obtained a 55.6% satisfaction in the transconjunctival group [16]. Although it is the gold standard to perform surgery in the post-inflammatory phase, which is the chronic phase, it is recommended to consider injection first for GO in the active phase. In this study, there was no statistically significant difference, but the prevalence period of non-responders tended to be longer: 3.07 ± 4.84 months, on average, for responders and 12.56 ± 23.21 months, on average, for non-responders. Free T4 prior to the injection was abnormal in 71.4% of responders and 25% of non-responders (*p* = 0.036), and the non-responder group, which was less responsive to the injection, may have more patients with chronic post-inflammatory phase than acute phase.

Due to their anti-inflammatory and immunosuppressive properties, glucocorticoids may be utilized in the treatment of UER in GO. According to Osaki et al., triamcinolone is relatively insoluble and slowly absorbed over several weeks, resulting in anti-inflammatory effects on the levator muscle and Mueller muscles, drooping eyelids by steroids, degenerative changes in musculature muscles, muscular tendon detachment from the tarsal plate, and atrophy of the Mueller muscles [12]. This study performed injections to treat UER regardless of thyroid condition following Uddin et al. who injected triamcinolone into the conjunctiva of 11 patients with chronic post-inflammatory phase [17]. Previous studies have indicated that injection of triamcinolone at the conjunctiva is less effective [17] and carries a risk of side effects such as elevated intraocular pressure, eyelid thinning, and eyelid discoloration during repeated injections [18,19,20]. However, in this study, no side effects were observed, and the majority of participants experienced positive effects from the injections. The observed outcome is attributed to the author’s technique of averting the eyelid, visually assessing the anatomical position, and administering the injection directly onto the eyelid plate.

Comparing the injection response to the upper eyelid in 95 patients studied by Osaki et al., the inflammatory GO group demonstrated the greatest results [12]. There are numerous studies in which four doses of 20 mg triamcinolone have been injected into the conjunctiva, as well as studies in which triamcinolone has been injected in the periocular area, with the edema around the eyes decreasing [7,8]. Xu et al. continued to inject triamcinolone into the conjunctiva and, after the injection, patients were separated into response and non-response groups based on whether the MRD1 decreased by more than 2 mm after the injection as in this study, with optimal results lasting at least six months [9]. The average UER improved by 2.31 mm, and the response group exhibited a short UER period prior to injection. According to a study by Chee et al., which demonstrated an improvement in UER in three out of four individuals with UER, mean improvement in MRD1 was 1.4 mm, with individuals with fibrotic muscle being unsuitable for injection [21]. In addition, Lee et al. found that individuals with severe UER prior to the triamcinolone injection did not show substantial improvement after the injection [22]. Chang et al., who verified levator muscles and Mueller muscles after the injection, discovered a significant thinning of the Mueller muscle, as well as a decrease in myosin light chain phosphorylation and α-smooth muscle actin levels [23].

The 5-FU has been used to treat cancer for a long time, and it has recently been found to be an effective anti-metabolic agent for managing dermal scars, particularly hypertrophic scars and keloids, by decreasing fibroblast proliferation and inhibiting collagen type 1 production. Ophthalmologists have used 5-FU, dose of 5 mg/mL (total dose of 15–50 mg/mL), to prevent scarring after trabeculectomy, 5-FU acting as an antimetabolite that can be applied during or after surgery to prevent the conjunctiva scarring down onto the sclera. A mechanism that inhibits the production of 5-FU fibrous substances in GO has been considered as one of the potential treatments for UER [24]. Additionally, injections of periocular hyaluronic acid have a duration of 6–12 months and are another option for treating UER in patients with GO. It has the effect of elongating the lower eyelid retractor and providing structural support to elevate the lid against the lower orbital rim. It may have a negligible effect, but it has the advantage of rectifying minor asymmetry prior to surgery [12].

Previously, GO patients with UER have been included in a study using BoNT-A, triamcinolone, dexamethasone, and 5-FU injections. After one month of multimodal injection therapy, MRD1 was found to have been corrected by more than 1 mm in 66.7% of patients [25].

In this study, an orbital CT scan conducted before the injection was able to distinguish this analysis from other studies, demonstrating EOM hypertrophy or fat hypertrophy in GO. In a recent study by Baixue et al., MRI and CT scans were performed on 3620 Chinese patients, the most frequently involved EOM on MRI was the inferior rectus (79.9%), and the superior recuts, medial rectus, and the lateral rectus were involved in this order [26]. This is comparable to the results of the 2009 study by Regensburg [27]. The area of EOM per cross-sectional area was larger in responders (*p* = 0.027), and the area of the lateral rectus was larger (*p* = 0.008). Even the least afflicted EOM exhibited muscle hypertrophy because of GO. This is a significant discovery, because the presence and absence of other comorbid diseases, such as IgG4 and pseudotumor, were validated and excluded, thereby confirming the EOM pattern of pure GO patients.

When comparing the ratios of EOM and fat per cross-sectional area, a high ratio of EOM indicated a responder, whereas a high ratio of fat indicated a non-responder. During the active stage of GO, EOM hypertrophy is known to be more prevalent [28]. The association between ocular protrusion and fat hypertrophy is widely recognized. In GO, the enlargement of fat and EOM is attributed to the abnormal accumulation of hyaluronic acid, tissue edema, and an increase in orbital adipose tissue volume [28]. In a recent study of repeated subconjunctival injections of triamcinolone acetonide in a volume of 0.5 mL (40 mg/mL), they found that that signal intensity ratio of the levator muscle was lower in the effective treatment group than in the non-effective treatment group on T1W-fat suppression contrast-enhanced 3.0 T MRI scans before and after the injection [29]. This showed that patients with poor treatment effect had more severe inflammation in the LPS muscle. No significant difference in T2W signal intensity ratio of the levator muscle between the effective group and non-effective group was found: the edema and inflammation of LPS resolved and the thickness decreased, but fibrotic complications in the Mueller muscle, levator, and surrounding tissue adhesions were still present in the non-effective group [29]. Consequently, the activity of a patient’s condition is in accordance with the degree of EOM hypertrophy, indicating a more favorable response to injection therapy. However, for chronic patients characterized by fat hypertrophy, it is anticipated that the treatment response may be less pronounced, prompting consideration of alternative treatment modalities in addition to injections.

Furthermore, the comparison of recent photographs with pre- and post-injection effects has proved to be valuable, as the patients continued to visit and receive monitoring at the ophthalmic outpatient clinic for GO. While previous studies have primarily focused on observing the effects one month after the injection, this study aimed to investigate the long-term effects by examining the progression not only after one month, but also up to the present time (with an average follow-up duration of 11 months). Nevertheless, the inclusion of patients who had undergone radiation therapy or received intravenous/oral steroid treatment prior to the injection has posed challenges to the assessment of the isolated effect of multimodal injections solely on the upper eyelid in this study. Additionally, the degree and location of the levator muscle thickening associated with the impaired function may be the key factors that explain the clinical manifestations of the eyelid [29]. The lateral portion of the levator, where the retraction is more dominant in GO, is relatively narrow in the orbital images for containing non-muscle tissue, and its individual variability is large. Therefore, measurement of the levator muscle thickening in terms of location and pattern could contribute to the understanding of the response to the anti-inflammatory agent or muscle paralytic drug. In order to predict the treatment effect for UER, studies using high-resolution MRI imaging are needed. Regarding the subtype of BoNT-A, further studies for optimal dosage and injection intervals could be conducted for the non-responders in the previous studies.

This study has the following limitations. It is essentially a retrospective study; therefore, only patients who had received orbital computed tomography before the treatment were enrolled. The number of cases is relatively small and the study lacks validation cases. Prospective studies with a slarge sample size and longer follow-ups in multicenter trials would be needed to provide long-term outcomes with limited side effects in the future.

## 5. Conclusions

In conclusion, a multimodal injection method of corticosteroids, 5-FU, and BoNT-A is both safe and efficient, especially in UER patients with hyperthyroidism and an elongated IPF. It significantly improves patients’ periocular conditions and effectively minimizes potential side effects, such as severe eyelid drooping, orbicularis oculi muscle paralysis-related lagophthalmos, increased intraocular pressure, and Mueller or eyelid muscle atrophy. Additionally, an increase in EOM cross-sectional area on CT, up to 150 mm^2^, may serve as a useful positive indicator for the use of multimodal injections in UER with GO. Consequently, the synergistic therapy using the lower dose of BoNT-A (2.5 units) and steroids (4 mg triamcinolone, 0.5 mg dexamethasone) seems to be effective in suppressing long-term fibroblastic proliferation by incorporating 5-FU and in obviating the need for repeated injections in UER patients with GO. It is necessary to take the EOM thickness in the orbit into consideration to address the UER, as we found that the ratio of EOM area to total orbital area was higher in the responders than in the non-responders.

## Figures and Tables

**Table 1:** Demographics of Graves’ ophthalmopathy (GO) patients according to the response to the multimodal injections of steroids, 5-FU, and botulinum neurotoxin A (BoNT-A) for upper eyelid retraction (UER).

	Responder (n = 14, 24 Eyes)	Non-Responder (n = 9, 13 Eyes)	Total (n = 23, 37 Eyes)	*p* Value
Sex (Male:Female)	1:13	2:7	3:20	0.557
Age (years)	41.71 ± 9.69	44.11 ± 11.30	44.0 ± 10.2	0.593
Graves’ disease duration (month)	2.36 ± 3.52	4.67 ± 6.73	3.26 ± 5.01	0.975
GO duration (month)	2.39 ± 2.00	2.17 ± 2.29	2.30 ±2.07	0.805
Free T4 (normal, 0.89~1.76 ng/dL)	4/14	6/9	10:12	0.036
TSH R Ab (normal, 0~1.75 U/L)	1/14	1/9	2:19	0.860
TS Ab (normal, 0~14%)	1/14	1/9	2:19	0.860
CAS	3.1 ± 0.7	2.9 ± 0.6	3.0 ± 0.8	0.539
NOSPECS	2.04 ± 1.04	2.77 ± 0.83	2.35 ± 1.06	0.078
Previous treatment (n = 12)				
Radiation therapy	2	1	3	
Methylprednisolone IV	2	1	3	
Prednisolone PO	2	1	3	
Thyroidectomy	1	0	1	
Side effect (diplopia, proptosis)	0	0	0	
Follow up periods (months)	12.71 ± 12.78	8.33 ± 9.61	11.0 ± 11.6	0.390

GO, Graves’ ophthalmopathy; CAS, clinical activity score; NOSPECS, no physical signs or symptoms, only signs, soft tissue involvement, proptosis, extraocular muscle signs, corneal involvement, and sight loss.

**Table 2:** Eyelid position and cross-sectional area of extraocular muscle measured by computed tomography (CT) scan of the Graves’ ophthalmopathy (GO) patients who were treated with multimodal injections of steroids, 5-FU, and botulinum neurotoxin A (BoNT-A) for upper eyelid retraction (UER).

	Responder (n = 14, 24 Eyes)	Non-Responder (n = 9, 13 Eyes)	Total (n = 23, 37 Eyes)	*p* Value
Before injection (mm)				
IPF	10.63 ± 1.08	9.88 ± 0.73	10.36 ± 1.03	0.039
MRD_1_	5.26 ± 0.85	4.62 ± 0.89	5.04 ± 0.91	0.067
MRD_2_	5.36 ± 0.76	5.25 ± 0.85	5.32 ± 0.78	0.838
Lid lag	3.48 ± 1.34	3.98 ± 2.10	3.64 ± 1.60	0.406
After injection (1 month, mm)				
IPF	10.07 ± 1.53	10.45 ± 1.08	10.20 ± 1.39	0.649
MRD_1_	4.34 ± 1.44	4.77 ± 1.06	4.49 ± 1.32	0.337
MRD_2_	5.72 ± 0.68	5.68 ± 1.10	5.71 ± 0.83	0.742
Lid lag	3.27 ± 1.23	4.49 ± 1.80	3.70 ± 1.55	0.025
Area (mm^2^)				
Orbit	637.4 ± 156.8	690.57 ± 85.76	668.97 ± 145.07	0.687
EOM	153.48 ± 17.95	132.00 ± 27.99	146.09 ± 23.83	0.027
Superior rectus m.	40.05 ± 11.50	36.18 ± 11.08	38.72 ± 11.33	0.434
Medial rectus m.	30.10 ± 7.27	28.27 ± 5.35	29.47 ± 6.64	0.696
Inferior rectus m.	36.43 ± 6.96	29.73 ± 12.20	34.13 ± 9.47	0.115
Lateral rectus m.	37.57 ± 9.71	29.82 ± 8.18	34.91 ± 9.82	0.008
Superior oblique m.	10.32 ± 3.15	9.78 ± 5.22	10.14 ± 3.84	0.772
Fat	487.81 ± 166.04	526.36 ± 71.82	501.06 ± 140.70	0.367
EOM/Orbit (%)	24.48 ± 6.21	19.30 ± 2.81	22.70 ± 5.80	0.003
Fat/Orbit (%)	72.01 ± 7.71	77.31 ± 3.41	73.84 ± 6.97	0.012

IPF: interpalpebral fissure, EOM: extra ocular muscle, m.: muscle.

## Data Availability

The data that support the findings of this study are available from the corresponding author upon reasonable request.
